# Standardization of a Molecular Technique for Human Papillomavirus Genotyping in a Public-Health Service

**DOI:** 10.7759/cureus.82524

**Published:** 2025-04-18

**Authors:** Sulani S De Souza, Mirian Helena H Abreu, Roberta M Paiva Ceribelli, Paula Cristina N da Silva, Gina C De Oliveira, Mariana M Roll

**Affiliations:** 1 Gynecology, Escola Superior de Ciencias da Saude, Brasilia, BRA; 2 Virology, Federal District Central Laboratory, Brasilia, BRA; 3 Lower Genital Tract Pathology, North Side Regional Hospital, Brasilia, BRA; 4 Gynecology, Euro-American University Center, Brasilia, BRA; 5 Lower Genital Tract Pathology, Taguatinga Regional Hospital, Brasilia, BRA; 6 Gynecology, Sobradinho Regional Hospital, Brasilia, BRA

**Keywords:** high-grade squamous intraepithelial lesion (hsil), hpv genotype, human papillomavirus infection, papanicolaou test, woman health

## Abstract

Objective: To standardize a molecular technique for genotyping human papillomavirus (HPV) and to evaluate its distribution and relationship with vaginal cytology.

Method: Women aged 25 years or older with altered cytology were selected from three public-health hospitals and underwent HPV genotyping by molecular biology. Samples were processed, stored, and subjected to extraction and amplification. Amplification was performed for 28 HPV types (19 of high-risk and 9 types of low-risk). The frequencies of the most prevalent HPV types and those with multiple genotypes, were calculated. The association between categorical variables was analyzed using the chi-square (χ^2^) and Fisher's exact test. Statistical significance was set at *p *< 0.05.

Results: The samples were divided into two groups: 1) without previous cervical treatment (177, 55%); and 2) with previous cervical treatment (142, 45%). The frequency of positive HPV was 126 (71%) and 67 (47%), respectively. The predominant high-risk HPVs were: 16, 58, 52 and 53; HPV53, HPV68 and HPV35 were associated with multiple infection in both groups. HPV16 and multiple infections were more prevalent between group age 25-35 years (*p* = 0,036; *p* = 0,034). High-grade intraepithelial lesions were associated with HPV16 in both groups (*p* = 0.001; *p* = 0.009) and with HPV53 in group 2 (*p* = 0.020). Cytology classified as atypical squamous cells of undetermined significance (ASCUS) (group 1) and negative for intraepithelial lesions and malignancy (NILM) (group 2) were associated with reduction of HPV16 (74.4%; 65.4%).

Conclusion: The two groups differed in the frequency of HPV types and the chance of single and multiple infections. High-grade intraepithelial lesions were associated with HPV16 in both groups.

## Introduction

Cervical cancer is the fourth most common cancer in women worldwide, and human papillomavirus (HPV) is the main causative agent of cervical cancer [1]. In Brazil, this cancer ranks third in importance, with an estimated 17,010 new cases each year for the biennium 2023-2025 and an estimated risk of 15.38 cases per 100,000 women [2]. In the Central-West region, it ranks third with an estimated risk of 16.66 cases per 100,000 women [2]. Cervical cancer has a significant social and economic impact, as it affects women at a relatively young age and has a high prevalence in less-developed regions of the world. The use of appropriate screening techniques is crucial for effective control and implementation of public-health interventions. In developed countries, mortality and incidence have been reduced by 80-90% with appropriate screening and population coverage vaccination [3].

According to the World Health Organization (WHO), to accelerate the elimination of the disease as a public-health problem, the following goals must be achieved by 2030: 1) 90% of girls must be fully vaccinated against HPV by age 15 years; 2) 70% of women must be screened with a high-performance test between the ages of 35 and 45 years; and 3) 90% of women identified with precursor lesions and cancer must receive treatment [4].

 In 2021, more than 5 million cervical-vaginal cytopathological examinations were performed using the national health system Sistema Unico de Saude (SUS); however, studies have shown that the sensitivity of this method is low and subject to various collection and processing problems. Primary screening using molecular tests for HPV appears to be a possible alternative, with better accuracy for early diagnosis. According to Teixeira et al. approximately 20% of cytology tests are performed on women under 25 years of age, and unfortunately, 60% of cervical cancers are diagnosed at advanced stages, even in the more developed regions of Brazil [5].

HPV is a circular, non-enveloped DNA virus that is transmitted through direct physical contact and can infect the skin and mucosa. It may be associated with the appearance of warts or cancer, and is classified into five types (alpha, beta, gamma, mu, and nu) according to the infected epithelium and survival mechanisms. There are more than 200 types of HPV, 15 of which have a high oncogenic risk. According to Small et al., the most frequent are 16, 18, 45, 31, 33, 52, 58, and 35 [6]. HPV can appear as single or multiple infections, as the host's immune response to infection varies. HPV16 is associated with approximately 70% of all invasive cervical cancer cases [7].

The Papanicolaou method has been used for cervical cancer screening for more than 50 years. It classifies cytological changes from I to V based on the loss of cytoplasmic maturation of cervical cells and cytological atypia (abnormal mitotic figures and changes in nuclear size and shape). These categories correspond, in the Bethesda system, to: (I) negative for intraepithelial lesions and malignancy (NILM); (II) atypical squamous cells of undetermined significance (ASCUS) and atypical squamous cells, cannot exclude high-grade squamous intraepithelial lesion (ASCH); (III and IIId) low-grade squamous intraepithelial lesions (LSILs); (IV) high-grade squamous intraepithelial lesions (HSILs); and (V) adenocarcinoma in situ (AIS). The classification between normal and borderline lesions can vary greatly among evaluators. Thus, calculating the sensitivity of this method is difficult and depends on who performs the collection and repetition of the exam at regular intervals [8]. It varies from 51% to 76% in patients with high-grade lesions. This technique is labor-intensive and requires mild, moderate, and severe grading of dyskaryosis. Histologically, LSIL corresponds to low-grade cervical intraepithelial neoplasia I (CIN I), while HSIL corresponds to intraepithelial neoplasia types II and III (CIN II and CIN III). Cytology identifies cervical lesions when the treatment is potentially curative [9]. The main problems are related to the collection and interpretation of findings. According to Bhatha et al., cytology has low sensitivity as a screening method, at approximately 53%, while HPV tests have 96.1% sensitivity despite their lower specificity (90.7% vs. 96.3%) [10].

The HPV test is a more objective method, and when well established, yields positive or negative results. It has high sensitivity for positive tests and a high negative predictive value for negative tests but low specificity. Thus, it safely identifies women who need to expand their investigation compared with those who can return to routine screening. It has been used: 1) in the triage of low-grade abnormal lesions found in cytology before referral to colposcopy; 2) in primary cervical cancer screening; 3) in cure evaluation after treatment; and 4) in the follow-up of women with positive cytology and/or HPV test results but without relevant histological lesions. When used as the primary test, a second cytology test is required for positive cases. Thus, a greater number of high-grade squamous intraepithelial neoplasia may be detected than that with conventional cytology [11,12].

Due to the high prevalence of HPV infection in the young population and the low prevalence in those over 50 years of age, HPV testing is recommended for women over 30 years of age [13,14]. It can be used to detect the presence of the virus before a lesion is established. According to a systematic review by Koliopoulos et al., this methodology reduced the loss of cases of histologically confirmed cervical intraepithelial neoplasia types 2 and 3 [15].

In this context, this study aimed to standardize the molecular genotyping technique and evaluate the association between abnormal cytological findings and the distribution of different HPV genotypes, especially the high-risk ones. The second reason is the concomitant occurrence of single and multiple infections.

## Materials and methods

Women aged 25 years or older were selected from three hospitals of the Secretaria de saude in the Federal District (Hospital Regional de Taguatinga (HRT), Hospital Regional da Asa Norte (HRAN), and Hospital Regional de Sobradinho (HRS)) who had an altered Pap smear from August 2022 to May 2024, classified as ASCUS, ASCH, LSIL, HSIL, AIS, and glandular cell atypia (AGC). All hospitals are part of the public healthcare network within the Federal District. The selection of these hospitals was deliberate to ensure representation from diverse geographical regions within the district, encompassing urban, suburban, and semi-rural populations. Methodologically, the study clearly defined two distinct patient groups: Group 1 included patients with altered cytology (Pap smear) who had not yet undergone cervical treatment procedures, while Group 2 comprised patients with altered cytology who had previously undergone treatment and were in a follow-up program, with samples collected only after the therapeutic procedure had been completed. The cervical material collection was performed by the doctor, using appropriate disposable material transferred to specific tubes, in liquid transport medium provided by the Laboratorio Central (LACEN) in the Federal District, and stored in a refrigerator (4°C to 8°C). The collected samples were sent to LACEN accompanied by a medical requisition with clinical indications and information for registration in the TrakCare system (Federal District) or Gerenciador de ambiente Laboratorial (GAL). This study was approved by the Human Research Ethics Committee of Escola Superior de Ciencias da Saude (ESCS) under number 6.510.136 (CAAE: 26454919.1.0000.5553).

Samples were processed, stored, and subjected to extraction and amplification. Genetic material extraction was performed in a 96-well plate with 150 μL of samples using a commercial kit from Zymo Research. The lysis technique used MagBinding Beads provided with the kit. Extraction was performed on Thermo Scientific equipment, and genetic material elution was performed in 100 μL.

Amplification was performed with the Anyplex^TM^ II HPV28 kit (Seegene, South Korea) using TOCE^TM^ technology to detect multiple pathogens in a single fluorescence channel. A multiplex assay allows simultaneous amplification, detection, and differentiation of nucleic acids from 19 of high-risk (16, 18, 26, 31, 33, 35, 39, 45, 51, 52, 53, 56, 58, 59, 66, 68, 69, 73, 82) and 9 types of low-risk (6, 11, 40, 42, 43, 44, 54, 61, 70) HPV, respectively, as well as an internal control (IC).

Anyplex II HPV28 detection was performed using either cyclic or endpoint melting temperature analysis. The IC was incorporated into the product as an endogenous process control to monitor nucleic acid extraction and verify possible polymerase chain reaction (PCR) inhibition and was co-amplified with target nucleic acids in clinical samples. The results were analyzed using the CFX96 Dx System software (CFX Manager Dx Software v3.1). According to the viral load represented by crosses, pathogen detection can be classified as absent (-) or three (+++), two (++), and one (+). The sensitivity detection limit was 50 copies/reaction.

The frequencies of the most prevalent HPV types and those with multiple genotypes, defined as two or more viruses, were calculated. The association between categorical variables was analyzed using the chi-square (χ^2^) and Fisher's exact test. Odds ratios (ORs) were calculated. The effect size was represented by Phi (ϕ), considering small, medium, and large when less than 0.1, 0.3, and 0.5 were detected, respectively [16]. Statistical significance was set at *p* < 0.05. Data was stored in Microsoft Excel. Statistical analyses were performed using IBM SPSS Statistics software (version 29.0).

## Results

A total of 319 women were selected, including 116 (36%), 23 (7%), and 180 (56%) from the HRAN, HRS, and HRT, respectively. The main regions assisted were: Samambaia 84 (26%), Taguatinga 54 (17%), Brasilia 33 (10%), Recanto das Emas 28 (9%), Sobradinho 21 (7%), Planaltina 18 (6%), Riacho Fundo 12 (4%), Gama 11 (3%), and Santa Maria 10 (3%). All underwent HPV genotyping screening.

Of the 319 participants, 177 (55%) were in group 1 (without cervical procedure) and 142 (45%) in group 2 (performed cervical procedure). An association was found between the presence of HPV types and genotyping group (χ^2^ (1) = 18.996, *p *< 0.001; ϕ = 0.244 (OR = 2.76, 95% confidence interval (CI) = 1.74-4.39)). The results are presented for groups 1 and 2. In total, 126 (71%) and 67 (47%) samples tested positive for HPV groups 1 and 2, respectively. Of these, 119 (67%) and 37 (21%), and 63 (44%) and 15 (11%) were positive for high and low risk, respectively, in the same groups. A total of 193 (61%) samples tested positive for HPV types. Of these, 108 (56%) and 85 (44%) tested positive for single and multiple HPV types, respectively (Table 1).

**Table 1:** Total, single, and multiple frequencies considering age group and indication for HPV genotyping n: number of samples; HPV: Human papillomavirus; HPV+: Human papillomavirus present; HPVsingle: Single human papillomavirus; HPVmulti: Multiple human papillomaviruses ^a^ *p *= 0.036; ^b^ *p* = 0.04; ^c^ *p *= 0.034

	Group 1				Group 2			
Age Range (Years)	HPV+ (n = 126)	HPVsingle (n = 65)	HPVmulti (n = 61)	Total Samples (n = 177)	HPV+ (n = 67)	HPVsingle (n = 43)	HPVmulti (n = 24)	Total Samples (n = 142)
25-35	33 (84.62) ^a^	14 (35.90)	19 (48.72) ^c^	39 (22.03)	16 (43.24)	10 (27.03)	6 (16.22)	37 (26.06)
35-45	50 (74.63)	31 (46.27) ^b^	19 (28.36)	67 (37.85)	21 (44.68)	15 (31.91)	6 (12.77)	47 (33.10)
45-55	22 (62.86)	12 (34.29)	10 (28.57)	35 (19.77)	16 (43.24)	10 (27.03)	6 (16.22)	37 (26.06)
55-65	16 (55.17)	6 (20.69)	10 (34.48)	29 (16.38)	10 (62.50)	6 (37.50)	4 (25.00)	16 (11.27)
>65	5 (71.43)	2 (28.57)	3 (42.86)	7 (3.95)	4 (80.00)	2 (40.00)	2 (40.00)	5 (3.52)

The median ages were 42 and 43 years, lower limits were 25 and 26 years, and upper limits were 74 and 76 years for group 1 and group 2, respectively. The 319 participants were divided into five age groups: 1) 25-35 years (76 (24%)); 2) 36-45 years (114 (36%)); 3) 46-55 years (72 (22%)), 4) 56-65 years (45 (14%)), and 5) > 65 years (12 (4%)). The age group of 35-45 years predominated for both groups, as shown in Table 1. The detection of HPV positivity and multiple infections was associated with the 25-35 age group (χ^2^ (1) = 4.398, *p*=0.036; ϕ = 0.158; χ^2^ (1) = 4.50, *p* = 0.034; ϕ = 0.159). Single infection was associated with the second age group (χ^2^ (1) = 4.227, *p *= 0.04; ϕ = 0.155). There was association between age 25-35 years and HPV16 in group 1 (χ^2^ (1) = 4.398, *p* = 0.036; ϕ = 0.158). This group of women had a 3.30 higher risk of HPV16 (95% CI: 1.5-7.17) (Table 2).

**Table 2:** HPV types by age group and indication for genotyping HPV: Human papillomavirus *p = 0.002

	Group 1					Group 2				
Type	25-35 years (n = 39)	36-45 years (n = 67)	46-55 years (n = 35)	56-65 years (n = 29)	>65 years (n = 7)	25-35 years (n = 37)	36-45 years (n = 47)	46-55 years (n = 37)	56-65 years (n = 16)	>65 years (n = 5)
HPV16	16 (40.00)*	14 (35.00)	4 (10.00)	5 (12.5)	1 (2.5)	4 (18.18)	8 (36.36)	4 (18.18)	5 (22.73)	1 (4.55)
HPV18	1 (9.09)	6 (54.55)	3 (27.27)	1 (9.09)	0 (0.00)	0 (0.00)	2 (50.00)	1 (25.00)	1 (25.00)	0 (0.00)
HPV31	3 (30.00)	4 (40.00)	1 (10.00)	1 (10.00)	1 (10.00)	0 (0.00)	1 (33.3)	1 (33.3)	1 (33.4)	0 (0.00)
HPV33	2 (22.22)	2 (22.22)	3 (33.33)	1 (11.11)	1 (11.11)	0 (0.00)	1 (50.00)	0 (0.00)	1 (50.00)	0 (0.00)
HPV35	3 (30.00)	4 (40.00)	2 (20.00)	1 (10.00)	0 (0.00)	1 (20.00)	0 (0.00)	3 (60.00)	0 (0.00)	1 (20.00)
HPV39	2 (40.00)	2 (40.00)	1 (20.00)	0 (0.00)	0 (0.00)	3 (100.00)	0(0.00)	0 (0.00)	0 (0.00)	0 (0.00)
HPV45	1 (20.00)	3 (60.00)	1 (20.00)	0 (0.00)	0 (0.00)	0 (0.00)	0 (0.00)	1 (100)	0 (0.00)	0 (0.00)
HPV51	1 (20.00)	2 (40.00)	1 (20.00)	1 (20)	0 (0.00)	2 (100.00)	0 (0.00)	0 (0.00)	0 (0.00)	0 (0.00)
HPV52	6 (33.33)	8 (44.4)	2 (11.11)	2 (11.11)	0 (0.00)	1 (16.67)	3 (50.00)	2 (33.33)	0 (0.00)	0 (0.00)
HPV56	1 (20.00)	1 (20.00)	0 (0.00)	2(40.00)	1 (20.00)	0 (0.00)	0 (0.00)	1 (25.00)	2 (50.00)	1 (25.00)
HPV58	5 (25.00)	8 (40.00)	5 (25.00)	1 (5.00)	1 (5.00)	3 (42.86)	3 (42.86)	0 (0.00)	0 (0.00)	1 (14.29)
HPV59	0 (0.00)	1 (50.00)	1 (50.00)	0 (0.00)	0 (0.00)	1 (25.00)	1 (25.00)	2 (50.00)	0 (0.00)	0 (0.00)
HPV66	0 (0.00)	1 (25.00)	3 (75.00)	0 (0.00)	0 (0.00)	1 (50.00)	0 (0.00)	1 (50.00)	0 (0.00)	0 (0.00)
HPV68	1 (20.00)	2 (40.00)	1 (20.00)	1 (20.00)	0 (0.00)	0 (0.00)	3 (60.00)	2 (40.00)	0 (0.00)	0 (0.00)
HPV26	1 (100.00)	0 (0.00)	0 (0.00)	0 (0.00)	0 (0.00)	0 (0.00)	0 (0.00)	0 (0.00)	0 (0.00)	0 (0.00)
HPV53	1 (7.69)	5 (38.46)	3 (23.08)	2 (15.38)	2 (15.38)	0 (0.00)	2 (40.00)	1 (20.00)	1 (20.00)	1 (20.00)
HPV69	0 (0.00)	0 (0.00)	3 (75.00)	1 (25.00)	0 (0.00)	2 (100.00)	0 (0.00)	0 (0.00)	0 (0.00)	0 (0.00)
HPV73	2 (66.67)	1 (33.33)	0 (0.00)	0 (0.00)	0 (0.00)	2 (100.00)	0(0.00)	0 (0.00)	0 (0.00)	0 (0.00)
HPV82	2 (28.57)	3 (42.86)	0 (0.00)	2 (28.57)	0 (0.00)	0 (0.00)	0 (0.00)	0 (0.00)	1 (100.00)	0 (0.00)

All investigated HPV types were identified in this sample, except for type 11, including 19 high-risk HPVs (16, 18, 26, 31, 33, 35, 39, 45, 51, 52, 53, 56, 58, 59, 66, 68, 69, 73, 82) and 8 low-risk HPVs (6, 40, 42, 43, 44, 54, 61, 70). Type 16 (33%) predominated, followed by 58 (16%), 52 (15%), 53 (11%), 18 (9%), 31 (8%), and 35 (8%) for group 1, and type 16 (44%), 58 (14%), 52 (12%), 53 (10%), 35 (10%), and 68 (10%) for group 2, considering high risk. For low risk, types 6 (50%) and 54 (50%) predominated for group 1, and 42 (33%), 44 (33%), and 54 (33%) for group 2.

Table 3 shows HPV types related to single or multiple infections for analyzed groups. For group 1, there was a significant association between HPV53 (Fischer's exact test,* p* < 0.001, ϕ = 0.343), HPV58 (χ^2^(1) = 12.607, *p* < 0.001, ϕ = 0.267), HPV68 (Fischer's exact test, *p *= 0.004; ϕ = 0.235), HPV51 (Fischer's exact test,* p* = 0.004; ϕ = 0.235), HPV31 (Fischer's exact test, *p* = 0.003; ϕ = 0.234), HPV52 (χ^2^(1) = 9.200,* p *= 0.002, ϕ = 0.228), HPV35 (Fischer's exact test, *p* = 0.033; ϕ = 0.183), HPV16 (χ ^2^(1) = 5.523, *p* = 0.019; ϕ = 0.177) and multiple infections. For group 2, there was a significant association between HPV16 (χ^2^(1) = 17.713, *p* < 0.001; ϕ = 0.353) and HPV58 (Fischer's exact test, *p* = 0.027; ϕ = 0.204) and single infections. OR analysis showed that the presence of HPV16 and HPV58 increased the chance of single infections by 7.04 (95% CI: 2.61-18.98) and 6.38 (95% CI: 1.18-34.32) times, respectively. There was a significant association between HPV53 (Fischer's exact test, *p* < 0.001; ϕ = 0.424), HPV59 (Fischer's exact test, *p* < 0.001; ϕ = 0.378), HPV73 (Fischer's exact test, p = 0.028; ϕ = 0.265), HPV56 (Fischer's exact test, *p* < 0.015; ϕ = 0.264), HPV68 (Fischer's exact test, *p* = 0.034; ϕ = 0.220), HPV35 (Fischer's exact test, *p* = 0.034; ϕ= 0.220) and multiple infections.

**Table 3:** Number of HPV types and indication for genotyping HPV: Human papillomavirus; HPVsingle: Single human papillomavirus; HPVmulti: Multiple human papillomaviruses; NS: Not significant

	Group 1				Group 2			
Type	HPVsingle	p	HPVmulti	p	HPVsingle	p	HPVmulti	p
HPV16	20 (50.00)	NS	20 (50.00)	0.019	15 (68.18)	<0.001	7(31.82)	NS
HPV18	4 (36.36)	NS	7 (63.64)	NS	2 (50.00)	NS	2 (50.00)	NS
HPV31	2 (20.00)	NS	8 (80.00)	0.003	2 (66.67)	NS	1 (33.33)	NS
HPV33	4 (44.44)	NS	5 (55.56)	NS	1 (50.00)	NS	1 (50.00)	NS
HPV35	3 (30.00)	NS	7 (70.00)	0.033	2 (40.00)	NS	3 (60.00)	0.034
HPV39	1 (20.00)	NS	4 (80.00)	NS	2 (66.67)	NS	1 (33.00)	NS
HPV45	1 (20.00)	NS	4 (80.00)	NS	0 (0.00)	NS	1 (100.00)	NS
HPV51	0 (0.00)	NS	5 (100.00)	0.004	2 (100.00)	NS	0 (0.00)	NS
HPV52	6 (33.33)	NS	1 2 (66.67)	0.004	3 (50.00)	NS	3 (50.00)	NS
HPV56	1 (20.00)	NS	4 (80.00	NS	1 (25.00)	NS	3(75.00)	0.015
HPV58	6 (30.00)	NS	14 (70.00)	<0.001	5 (71.43)	0.027	2 (28.57)	NS
HPV59	1 (50.00)	NS	1 (50.00)	NS	0 (0.00)	NS	4 (100.00)	<0.001
HPV66	2 (50.00)	NS	2 (50.00)	NS	1 (50.00)	NS	1 (50.00)	NS
HPV68	0 (0.00)	NS	5 (100.00)	0.004	2 (40.00)	NS	3 (60.00)	0.034
HPV26	0 (0.00)	NS	1 (100.00)	NS	0 (0.00)	NS	0 (0.00)	NS
HPV53	1 (7.69)	NS	12 (92.31)	<0.001	0 (0.00)	NS	5 (100.00)	<0.001
HPV69	3 (75.00)	NS	1 (25.00)	NS	1 (50.00)	NS	1 (50.00)	NS
HPV73	1 (33.33)	NS	2 (66.67)	NS	0 (0.00)	NS	2 (100.00)	0.028
HPV82	3 (42.86)	NS	4 (57.14)	NS	0 (0.00)	NS	1 (100.00)	NS

Of the 193 cases, 108 (56%), 56 (29%), 19 (10%), 7 (4%), and 3 (1%) were positive for one, two, three, four, and more than five HPV types, respectively. Of the identified types, 44 (35%), 88 (70%), and 46 (36%), and 21 (31%), 49 (73%), and 9 (13%) were observed semi-quantitatively with +/3+, 2+/3+, and 3+/3+ for group 1 and group 2, respectively.

Table 4 shows the distribution of the 319 selected samples according to cytological type and the number of HPV within the groups. In group 1, there was a predominance of ASCUS 60 (34%) and HSIL 48 (27%) cytological types; HSIL was significantly associated with HPV16 (χ^2^(1) = 28.270, *p *= 0.001; ϕ = 0.400). Women with HSIL and HPV16 had 7.06-fold increases (95% CI: 3.27-13.26) and ASCUS with HPV16 had reduction of 74.4% (OR 0.256; 95% CI: 0.074-0.88). However, for group 2, there was a predominance of NILM 89 (63%) and HSIL 20 (14%) cytological types; ASCH was significantly associated with HPV58 (Fischer's exact test, *p *= 0.013; ϕ = 0.282); HSIL with HPV53 (Fischer's exact test, *p* = 0.020; ϕ = 0.252) and with HPV16 (χ^2^(1) = 6.766, *p* = 0.009; ϕ = 0.218). Women with NILM with HPV16 had reduction of 65.4% in infections (OR 0.346; 95% CI: 0.136-0.878).

**Table 4:** Cytological types, indication for genotyping, and prevalent HPV types LSIL: Low-grade squamous intraepithelial lesion; ASCUS: Atypical squamous cells of undetermined significance; ASCH: Atypical squamous cells, cannot exclude high-grade squamous intraepithelial lesion; HSIL: High-grade squamous intraepithelial lesion; AGC: Atypical glandular cells; AIS: Adenocarcinoma in situ; NILM: Negative for intraepithelial lesion and malignancy; HPV: Human papillomavirus ^a^ *p* < 0.001; ^b^ *p *= 0.009; ^c^ *p *= 0.013; ^d^ *p* = 0.020

Cytological types	Gruop 1				Cytological types	Gruop 2			
	HPV16	HPV58	HPV52	HPV53		HPV16	HPV58	HPV52	HPV53
AGC	1 (2.50)	1 (5.00)	0 (0.00)	1 (8.00)	AGC	1 (4.00)	0 (0.00)	0 (0.00)	0 (0.00)
ASCUS	3 (7.50)	3 (15.00)	5 (28.00)	1 (8.00)	ASCUS	0 (0.00)	0 (0.00)	2 (33.00)	0 (0.00)
ASCH	8 (20.00)	7 (35.00)	8 (44.00)	5 (38.00)	ASCH	3 (14.00)	3 (43.00)^c^	0 (0.00)	0 (0.00)
HSIL	24 (60.00)^a^	4 (20.00)	5 (28.00)	5 (38.00)	HSIL	7 (32.00)^b^	0 (0.00)	1 (17.00)	3 (60.00)^d^
LSIL	1 (2.50)	4 (20.00)	0 (0.00)	1 (8.00)	LSIL	0 (0.00)	1 (14.00)	1 (17.00)	0 (0.00)
AIS	3 (7.50)	1 (5.00)	0 (0.00)	0 (0.00)	AIS	2 (9.00)	0 (0.00)	0 (0.00)	0 (0.00)
NILM	0 (0.00)	0 (0.00)	0 (0.00)	0 (0.00)	NILM	9 (41.00)	3 (43.00)	2 (33.00)	2 (40.00)
Total	40	20	18	13	Total	22	7	6	5

## Discussion

HPV genotyping is an important methodology to be included in the diagnostic arsenal for detecting precursor lesions of cervical cancer, as it is objective, extends screening time, and helps to identify positive cases early, preventing severe and difficult-to-control forms. With Covid-19, technological advancements have expanded the use of molecular biology techniques for diagnosis.

In this cross-sectional observational study, two groups were evaluated: 1) those without procedure, and 2) those who underwent cervical procedure. For group 1, the main findings were: a) Positive HPV frequency of 126 (71%)-the predominant HPV types were 16, 58, 52, and 53 for high risk and 6 and 54 for low risk; b) HPV53 showed a significant association with multiple infections of medium effect size, followed by HPV58, HPV68, HPV51, HPV31, HPV52, HPV35 and HPV16; c) cytology classified as HSIL was associated with an increase in HPV16 (medium effect size), while ASCUS was associated with a reduction in HPV16; and d) semi-quantitative predominance of infection with 2+ and 3+ was observed.

For group 2: a) Positive HPV frequency of 67 (47%)-the predominant HPV types were 16, 58, 52, and 53 for high risk and 42, 44, and 54 for low risk; b) HPV16 and HPV58 showed a significant association with single infections, while HPV53 and HPV59 with medium effect size, followed by HPV73, HPV56, HPV68, HPV35 with multiple infections; c) cytology classified as HSIL was associated with HPV16 and HPV53, while ASCH was associated with HPV58 and NILM associated with a reduction in HPV16; and d) semi-quantitative predominance of infection with 1+ and 2+ was observed.

Our results align with Camara et al., who reported 62% HPV positivity in women with altered cytology, with types 16, 58, 31, 53, 18, and 33 being the most frequent among the 11 HPV types studied [17]. Miranda et al. reported 44 (11%) cases in 399 women with normal cytology, for whom 12 types were tested, with types 16, 83, and 66 being the most frequent high-risk types [18]. Both studies were conducted using samples from the Federal District. Discrepancies with these studies can be explained by the different methodologies and sample selection.

According to Bruni et al. the global prevalence of HPV for the five continents was estimated at 11.7% (95% CI: 11.6-11.7), although Latin America had a prevalence of 16.1% [19]. The most frequent HPV types were 16, 18, 52, 31, and 58. There was agreement in relation to the types identified in this study. Type 16 was the most prevalent, consistent with studies from all Brazilian regions [20,21].

Colpani et al. reported a total HPV prevalence of 28.41% (95% CI: 22.71-28.32) and a high-risk HPV prevalence of 17.65% (95% CI: 14.80-20.92) [22]. However, they highlighted that in high- and low-risk populations, the total prevalence was 38.01% (95% CI: 25.90-51.82) and 24.11% (95% CI: 21.50-26.930), respectively. Thus, the sample selected for this study did not accurately describe the study population, as it was biased. Differences in prevalence may be related to the complex interactions among the virus, environment, and individual, especially in this study with sample selection.

Infection occurred with one HPV type and multiple HPV types in 108 (56%) and 85 (44%) of the samples, respectively. Co-detection occurred for high-risk HPV, low-risk HPV, or high- and low-risk HPV together. Kops et al. reported 1,279 (33%) cases of multiple infections in a national multicenter study; however, the participants were aged between 16 and 25 years [23]. Luo et al. in a study of 20,059 women, observed a bimodal distribution in age extremes of HPV, and those with HSIL and LSIL had multiple types of infections, as did those with ASCUS, although the latter was not statistically significant, and the most prevalent type associated with HSIL was HPV16 in women undergoing screening [24]. Kim et al. analyzed 1,967 women, with multiple infections in 236 (11.9%), and observed that HPV53 was the most prevalent type in multiple infections, followed by HPV16, HPV58, and HPV52, with HSIL being the most common cytological type [25]. In this study, the age group 25-35 years showed more HPV16 and multiple infections; HSIL was associated with HPV16 in both groups and with HPV53 in group 2. The prevalence of HPV types associated with multiple infections is consistent with that reported by Kim et al. However, in this study, two groups with specific characteristics were observed, the sample size was small, and the participating women had a history of altered cytology. The lack of detection of this bimodal pattern draws attention to the high detection of genotypes at an early age and a population with a high overall HPV prevalence.

In a study of 294 women with altered cervical cytology, Oyervides-Muñoz et al. observed multiple infections in 105 (59%) and a high viral load among those with persistent infections [26]. Women with normal cytology had a lower HPV16 viral load than those with LSIL. These authors reinforced the findings regarding viral load observed in this study, where participants predominantly had a viral load of 2+ and 3+, considering a total of 3+, especially in group 1. If the genetic material amplification process is divided into three stages, the participants begin to show a positive load from the beginning for some, and others in the second third of the process. For group 2 with viral loads of 2+ and 1+, the virus was detected only in the second and last thirds of the process. However, further studies are required to confirm these findings.

The findings of the present study have several important implications. First, the status of the incorporation of molecular tests for genotyping by the Unified Health System is published in the Official Diary [27]. This was the first public central laboratory to validate this genotyping methodology. These results demonstrate the importance of identifying HPV types, whether in group 1 or group 2 of women, who can be guided with more property using this information, as recommended by Carvalho et al., who published a flowchart for screening after HPV genotyping [28].

This study has several strengths, including sample diversity, validation of the method for performing HPV genotyping by molecular biology with a kit included in recommendation number 878 for molecular testing and detection of HPV and cervical cancer screening, for the population of the Federal District, and assistance in following women who have already undergone some procedure [29].

However, we must recognize the limitations of this study. First, the cross-sectional nature of this study prevents causal inferences. Additionally, there are biases in selecting the tested sample, subdividing it into two groups, and covering the regions for genotyping. This study does not represent a general screening population; rather, it is specifically focused on women already identified with altered oncotic cytology. The primary aim was the standardization of HPV genotyping methods in patients with positive oncotic cytology. These limitations should be considered when interpreting the results.

Based on the results, we suggest several directions for future research. First, longitudinal studies are conducted to explore causal relationships. Additionally, future studies should include a larger sample size for the groups, evaluate other relationships, assess the role of viral load, and expand the coverage area. Future investigations can help clarify the relationship between HPV types and those in the era after vaccination.

## Conclusions

The two groups differed in the frequency of HPV types and the chance of single and multiple infections. High-grade intraepithelial lesions were associated with HPV16 in both groups. This study demonstrates how much additional information can be obtained regarding the presence of HPV types and suggests the early performance of low-complexity procedures that can change the life history of each woman involved. Despite these limitations, the findings provide new information on the prevalent HPV types in parts of the Federal District and suggest that this is an initial milestone for implementing this diagnostic method for women with the intention of promoting their health. The results highlight the importance of this methodology as a diagnostic tool, as it can be used to refine screening strategies, prioritize women at an increased risk of developing high-grade lesions, monitor those in outpatient follow-up, and detect possible recurrences in those with a history of altered cytology.
